# Early Outcomes of Transhiatal Esophagectomy: Experience From an Oncology Center in Nepal

**DOI:** 10.7759/cureus.108337

**Published:** 2026-05-06

**Authors:** Sunil Dhakal, Nimesh Bista, Bibek Neupane, Saurav Karki, Deepak Sharma

**Affiliations:** 1 Gastrointestinal Surgery, BP Koirala Memorial Cancer Hospital, Bharatpur, NPL; 2 Surgery, Nepal Army Institute of Health Sciences, Shree Birendra Hospital, Kathmandu, NPL; 3 Surgical Gastroenterology, Maharajgunj Medical Campus Institute of Medicine, Tribhuvan Teaching Hospital, Kathmandu, NPL

**Keywords:** complications, early outcomes, feasibility assessment, lymph node dissection, transhiatal esophagectomy

## Abstract

Background: Esophagectomy is a highly morbid surgical procedure. Both transhiatal and transthoracic esophagectomies are performed for carcinoma of the lower third of the esophagus and gastroesophageal (GE) junction. Compared with transthoracic esophagectomy, transhiatal esophagectomy (THE) is considered relatively less morbid, as thoracotomy is not performed. However, THE is associated with less lymph node retrieval compared to the transthoracic procedure. This study was conducted to assess the feasibility, safety, and early postoperative outcomes of THE.

Methods: This study was conducted by a retrospective review of 10 patients who underwent THE between July 2022 and July 2024 in the Department of Gastrointestinal Surgery, B.P. Koirala Memorial Cancer Hospital, Bharatpur, Nepal. The preoperative, intraoperative, and postoperative variables were analyzed. The postoperative complications within 30 days were evaluated, and the Comprehensive Complication Index (CCI) was calculated.

Results: The most common indication for THE was carcinoma of the GE junction, observed in eight patients (80%), while the remaining patients had carcinoma of the lower third of the esophagus. Adenocarcinoma was the histological subtype in all patients. The mean duration of surgery was 267 ± 53.23 minutes, with a mean intraoperative blood loss of 380 ± 112.7 mL. There was no intraoperative bleed requiring thoracotomy. Intraoperatively, six patients (60%) required chest tube placement due to pleural breach. Postoperatively, the mean CCI was 18.65 ± 11.91, and major complications (CCI > 23) occurred in two patients (20%). Vocal cord paresis was the most common complication, occurring in six patients (60%) who recovered within the third to fourth postoperative day, followed by pneumonia in five patients (50%) and surgical site infection in five patients (50%). One patient developed an anastomotic leak that was managed conservatively. On histopathological examination, all resection margins were negative (R0 resection), with a mean lymph node retrieval of 14 ± 5.12.

Conclusion: THE is a feasible surgery as an initial approach in a dedicated center. It is associated with minimal major complications and low mortality. However, lymph node retrieval remains below the recommended standard. Therefore, a large-scale prospective study is warranted to validate these findings in our population.

## Introduction

Esophagectomy is the definitive treatment for carcinoma of the esophagus and Siewert type I and II gastroesophageal (GE) junction tumors [1]. The main surgical approaches include transhiatal esophagectomy (THE), McKeown, and Ivor-Lewis esophagectomy. Both McKeown's and Ivor-Lewis’s procedures require thoracotomy for transthoracic esophageal dissection, whereas THE involves abdominal gastric mobilization followed by transhiatal dissection of the lower esophagus without thoracotomy and reconstruction with a cervical esophagogastric anastomosis [2]. Compared to THE, transthoracic techniques allow better tumor visualization and more extensive lymph node retrieval. However, THE is associated with decreased morbidity and mortality [3].

Early complications are defined as those occurring within 30 days of surgery and reflect the quality, safety, and feasibility of the procedure. The overall incidence of complications after esophagectomy has been reported to be as high as 59%, with pneumonia being the most common, followed by atrial dysrhythmia, anastomotic leak, chyle leak, conduit necrosis, and recurrent laryngeal nerve (RLN) injury [4]. THE remains an important surgical option, although considered a less morbid procedure; its early outcomes require further evaluation. Thus, the aim of this study is to assess the feasibility, safety, and early postoperative outcomes with oncological adequacy of THE in our center.

## Materials and methods

This retrospective study analyzed data from the first 10 THE performed for carcinoma of the GE junction and lower esophagus. The study period was from July 2022 to July 2024 and was conducted in the Department of Gastrointestinal Surgery, B.P. Koirala Memorial Cancer Hospital, Bharatpur, Nepal. The Institutional Review Committee of B.P. Koirala Memorial Cancer Hospital issued approval for the study (approval number: 37/081/082). All patients had undergone esophagogastroscopy, and a preoperative diagnosis was established through tissue biopsy. Contrast-enhanced computed tomography (CECT) of the chest, abdomen, and pelvis was performed for staging. Patients with stage I tumors underwent upfront surgery, while those with higher-stage tumors were planned for neoadjuvant therapy. Upfront surgery was performed in patients who declined neoadjuvant therapy. 

Surgical aspect

All surgeries were performed via an open approach using an upper midline incision. A standard two-field lymphadenectomy was carried out in all cases. The gastric conduit was utilized for esophageal reconstruction in all patients. Anastomosis was created as a hand-sewn end-to-side esophagogastric cervical anastomosis. A feeding jejunostomy was placed in all cases, and feeding was initiated on the first postoperative day. A chest tube was inserted whenever there was suspicion of a pleural breach. An abdominal drain was placed in all patients.

Outcome analysis

Early outcomes, defined as events occurring within 30 days postoperatively, were analyzed [4]. Complications were evaluated using the Comprehensive Complication Index (CCI), with scores greater than 23 as major complications and <23 as minor complications [5]. Pulmonary complications, including pneumonia and pleural effusion, were diagnosed based on clinical presentation, radiological findings (chest X-ray or CECT), and sputum culture results. Cardiac complications, such as arrhythmias and heart failure, were diagnosed through assessment of cardiac enzymes, electrocardiographic changes, and consultation with a cardiologist. RLN palsy was suspected in patients presenting with hoarseness of voice. A chyle leak was diagnosed when postoperative drain output was milky or turbid, with fluid triglyceride levels exceeding 110 mg/dL [6]. The oncological adequacy of surgery was assessed from the histopathology reports, using circumferential R0 resection status and lymph node yield as key parameters.

Data analysis

Continuous data were analyzed and expressed as mean ± standard deviation (SD) or median with interquartile range (IQR) as appropriate. Categorical variables were presented as frequency and percentage. Statistical analysis was done using IBM SPSS Statistics for Windows, Version 25.0 (IBM Corp., Armonk, New York, United States). 

## Results

A total of 10 patients who underwent THE were included. The mean age at presentation was 60.7 ± 8.48 years, with an equal distribution of males and females (male-to-female ratio 1:1). Comorbidities were present in five patients (50%), with hypertension in three (30%) and diabetes mellitus in two (20%). Smoking history was reported in three patients (30%). All patients were diagnosed with adenocarcinoma. Among them, four patients (40%) had Siewert type I, four (40%) had Siewert type II, and two (20%) had carcinoma of the lower one-third of the esophagus. Preoperatively, neoadjuvant chemotherapy (NACT) was planned for seven patients (70%), of whom only four patients received NACT. The median carcinoembryonic antigen (CEA) level was 4±23.55, as the CEA level is variable among patients, with a minimum and maximum range documented in a patient of 1.4 and 78. The preoperative characteristics are mentioned in Table 1.

**Table 1:** Preoperative characteristics of the patients The median carcinoembryonic antigen (CEA) level was 4±23.55, as the CEA level is variable among patients, with a minimum and maximum range documented in a patient being 1.4 and 78.

Parameters	Details (N=10)
Age (years, mean ± SD)	60.7 ± 8.48
Male N (%)	5 (50%)
Female N (%)	5 (50%)
Smoker N (%)	3 (30%)
Diabetes, N (%)	2 (20%)
Hypertension, N (%)	3 (30%)
Chronic obstructive pulmonary disease (COPD) , N (%)	0
Cardiac disease, N (%)	0
Site of tumor	
Gastroesophageal junction (GEJ) Siewert type 1, N (%)	4 (40%)
GEJ Siewert type 2, N (%)	4 (40%)
Lower 1/3^rd^ esophagus, N (%)	2 (20%)
Type of tumor	
Adenocarcinoma, well to moderately differentiated, N (%)	7 (70%)
Adenocarcinoma, poorly differentiated, N (%)	3 (30%)
Squamous cell carcinoma, N (%)	0
Neoadjuvant chemotherapy N (%)	4 (40%)
Carcinoembryonic antigen (CEA; median ± SD)	4 ± 23.55
Hemoglobin (mean ± SD)	11.31± 2.10
Albumin (mean ± SD)	3.68 ± 0.57

The mean duration of surgery was 267±53.23 minutes with a mean blood loss of 380±112.7 mL. During hiatal dissection, a pleural breach was suspected in six (60%) patients, and chest tubes were placed accordingly. Isolated left pleural breach occurred in two patients (20%), and bilateral pleural breach in four patients (40%). The intraoperative characteristics are presented in Table 2.

**Table 2:** Intraoperative characteristics of the patients

Parameters	Details (N=10)
Duration of surgery (Mean ±SD) minutes	267± 53.23
Blood loss (mean ± SD) ml	380± 112.7
Blood transfused N (%)	3 (30%)
Inotropes used N (%)	1 (10%)
Conduit	
Stomach N (%)	10 (100%)
Colon N (%)	0
Feeding jejunostomy N (%)	10 (100%)
Pleural breach N (%)	6 (60%)
Isolated left pleural breach N (%)	2 (20%)
Bilateral pleural breach N (%)	4 (40%)
Chest tube placement N (%)	6 (60%)
Anastomosis	
Hand-sewn N (%)	10 (100%)
Stapled N (%)	0

Postoperatively, the mean duration of ICU stay was 3.2±3.11 days, with a median duration of hospital stay of 14±10.34 days. The first oral feeding was started at a median of 5±8.21 days. The earliest time for feeding was the third day, and in all patients, oral feeding was started within the seventh day. However, one patient who underwent conservation management had an anastomotic leak; oral feeding was started after 30 days. While all patients received feeding via jejunostomy starting on postoperative day (POD) 1. The mean CCI was 18.65±11.91, with two patients (20%) experiencing major complications. Pulmonary complications, specifically pneumonia, occurred in five (50%) patients, and surgical site infection occurred in five (50%) patients. Six (60%) patients developed temporary hoarseness, which resolved by POD 3-4. One patient had an anastomotic leak that healed with conservative management. There were no cases of chyle leak, cardiac complications, or 30-day mortality. The mean number of lymph nodes retrieved was 14 ± 5.12, with no margin positivity. Two patients (20%) were staged as stage I, both with carcinoma of the lower third of the esophagus. One patient (10%) was stage II, and the remaining seven patients (70%) were stage III. The postoperative characteristics are mentioned in Table 3.

**Table 3:** Postoperative characteristics The first oral feeding was started at a median of 5±8.21 days. The earliest time to feed was the third day, and in all patients, oral feeding was started within the seventh day. One patient had an anastomotic leak; oral feeding was started after 30 days.

Parameters	Details (N=10)
ICU stay (mean ± SD) days	3.2±3.11
Hospital stays (mean ± SD) days	14±10.34
First oral feeding (median ± SD) days	5±8.21
Comprehensive Complication Index (CCI; mean ±SD)	18.65±11.91
Major (CCI≥ 23), N (%)	2 (20%)
Minor (CCI<23), N (%)	8 (80%)
Mortality (30 days) N (%)	0
Anastomotic leak N (%)	1 (10%)
Pulmonary complications N (%)	5 (50%)
Chyle leak N (%)	0
Vocal cord paresis N (%)	6 (60%)
Surgical site infection (SSI) N (%)	5 (50%)
Lymph node (mean ±SD)	14±5.12
Margin positive N (%)	0
Histopathology	
Stage I	2 (20%)
Stage II	1 (10%)
Stage III	7 (70%)
Stage IV	0
Adjuvant therapy N (%)	5 (50%)

## Discussion

Multimodality treatment is currently the standard of care for esophageal and GEJ junction cancers. Neoadjuvant therapy is indicated in patients with locally advanced resectable disease, particularly those with clinical tumor-node-metastasis (TNM) stage T2-T4 or node-positive disease (N1-N3). Recent evidence from the phase III ESOPEC (Esophageal SOperative PErioperative Chemotherapy) trial demonstrated that perioperative chemotherapy with 5-fluorouracil, leucovorin, oxaliplatin, and docetaxel (FLOT) is an effective and preferred neoadjuvant strategy for adenocarcinoma of the esophagus and GE junction. In the present study, NACT was planned for seven patients with locally advanced resectable disease; however, only four patients received the treatment. Postoperatively, adjuvant therapy was advised for eight patients, but only five patients received it. This lower adherence to chemotherapy in our cohort may be attributed to poor economic compliance and loss to follow-up among patients in our population [7-8].

Prolonged duration of surgery has been associated with adverse postoperative outcomes, as longer operative time increases the risk of postoperative complications. Compared with transthoracic esophagectomy, THE is generally shorter, with operative duration reported to be approximately 100 minutes shorter. A large study involving 5,098 patients undergoing esophagectomy identified operative time thresholds associated with improved outcomes, with a cut-off of 333 minutes for THE and 422 minutes for Ivor-Lewis esophagectomy. The same study also reported that chronic obstructive pulmonary disease (COPD) was a significant factor associated with prolonged operative time in patients undergoing THE and was linked to poorer prognosis. In the present study, the mean duration of surgery was 267 ± 53.23 minutes, which is within the benchmark suggested by the study. Additionally, none of the patients had COPD, which may have contributed to the acceptable operative duration and favorable perioperative outcomes [9]. Initially, there was concern about excessive intraoperative hemorrhage, reported in 1.3%-3% of cases. After the introduction of long-angle clamps with direct clamping and ligation of periesophageal tissue, blood loss was reduced to less than 1%, with an average of 360 mL reported by Orringer MB et al. [10]. Further improvements were achieved using the transcervical endoscopic esophageal mobilization approach, reducing mean blood loss to 251 mL [11]. In our practice, we do not routinely use long clamps for periesophageal vessel control and perform blunt, blind dissection of the esophagus. This results in slightly higher blood loss from intramural vessels, averaging 380 ± 112.7 mL. There was no injury to the bronchial artery or azygos vein, which can cause severe bleeding necessitating conversion to thoracotomy. The incidence of pleural injury was noted intraoperatively in 60% of patients, requiring chest tube placement, which is similar to minimal invasive esophagectomy, where it has been reported to be 60.6% [12]. Pleural injury is frequently caused by blunt dissection along the right side of the esophagus, cervicopleural communication, and excision of pleura adherent to the tumor [13-14].

RLN injury is one of the most frequent complications following THE. The reported incidence ranges from 14% to 29% in published studies. RLN palsy occurs due to stretch, compression, or thermal injury during cervical or mediastinal dissection, leading to hoarseness and unilateral vocal cord paralysis. Previous studies have reported that approximately 30% of RLN injuries remain asymptomatic and undiagnosed, and, therefore, routine postoperative laryngoscopy evaluation has been recommended. In the present study, 60% of patients developed postoperative hoarseness, which improved spontaneously within three to four days, and laryngoscopy was not performed as symptoms gradually resolved. [15,16] Pulmonary complications following esophagectomy have been reported in 11% to 20% of patients. Among these, pneumonia is one of the most serious complications, with several recognized risk factors including aspiration secondary to RLN palsy, poor oral hygiene, COPD, and sarcopenia. In our study, the relatively high incidence of RLN injury may have contributed to aspiration and pneumonia observed in 50% of affected patients [15-16].

Anastomotic leak is a devastating complication after esophagectomy, associated with increased postoperative morbidity, mortality, and adverse long-term outcomes. The reported leak rate following esophagectomy ranges from 4% to 10% in the literature. In a meta-analysis by Hung PC et al., no significant difference in anastomotic leak rate was observed based on the type of esophagectomy performed. The gastric pull-up remains the conduit of choice for reconstruction after esophagectomy unless contraindicated, such as in cases of tumor extension into the stomach, corrosive gastric injury, previous gastric surgery, or failure of gastric conduit formation. The gastric conduit is primarily supplied by the right gastroepiploic vessels and is typically not supercharged. Recently, supercharged cervical anastomosis techniques have been described to improve conduit perfusion; however, current evidence suggests that this approach has not consistently reduced the incidence of anastomotic leak. Intraoperative indocyanine green (ICG) fluorescence imaging has been increasingly used to assess conduit perfusion and has been shown to reduce anastomotic leak rates in several studies [17]. In our study, supercharging of the conduit and ICG perfusion assessment were not performed, and the observed anastomotic leak rate was 10%, which is within the range reported in the literature.

On evaluation of the oncological adequacy of THE, the mean lymph node retrieval in our study is 14±5.12, which is lower than the number recommended by the National Comprehensive Cancer Network (NCCN), which is at least 15 lymph nodes [18]. A previous meta-analysis by Wolff CS et al. demonstrated that the mean lymph node retrieval in THE was nine compared with 18 in the Ivor-Lewis esophagectomy, with a statistically significant difference (p < 0.001) [19]. Similarly, a randomized controlled trial involving a large cohort of patients conducted by Mertens AC et al. showed that the transthoracic approach yielded a higher lymph node count, with 14 nodes retrieved in THE and 19 nodes in the transthoracic group (p < 0.001) [20]. However, improvements in lymph node retrieval have been reported with mediastinoscopy-assisted THE, as demonstrated by Masuda Y et al., where the mean lymph node yield was 18.6 [16]. In our study, all surgical margins (proximal and distal) were negative, as the tumors were located in the lower esophagus or GE junction, and resections were performed up to the cervical esophagus with cervical anastomosis. 

The limitations of this study are that this was a retrospective analysis conducted at a single center with a small sample size of only 10 patients.

## Conclusions

THE is a feasible surgery as an initial approach in a dedicated center. As it can be safely performed with an optimal duration, blood loss. It is associated with minimal major complications that can be managed with a conservative approach and hence is associated with low mortality. THE remains the choice of surgery for the initial approach; however, lymph node retrieval remains below the recommended standard. Therefore, a large-scale prospective study is warranted to validate these findings in our population.
